# Exploring the impact of cattle on human exposure to malaria mosquitoes in the Arba Minch area district of southwest Ethiopia

**DOI:** 10.1186/s13071-020-04194-z

**Published:** 2020-06-22

**Authors:** Melkam Abiye Zeru, Simon Shibru, Fekadu Massebo

**Affiliations:** 1 Department of Medical Parasitology, University of Gondar, Gondar, Ethiopia; 2 Department of Biology, Arba Minch University, Arba Minch, Ethiopia

**Keywords:** Cattle baited collection, Chano village, Host-seeking behaviour, Hourly biting rhythm, *Anopheles* mosquitoes

## Abstract

**Background:**

The success of indoor interventions that target mosquitoes for malaria control is partially dependent on early evening and outdoor biting behaviours of mosquito vectors. In southwest Ethiopia, people and cattle live in proximity, which calls to investigate whether the presence of cattle increase or decrease bites from malaria mosquito vectors. This study assessed both host-seeking and overnight activity of malaria mosquito vectors given the presence or absence of cattle in Chano Mille village, Arba Minch district, Ethiopia.

**Methods:**

*Anopheles* species density and activity time was compared when a calf was: (i) placed inside; (ii) 1 m away from; or (iii) absent from a tent with a human volunteer resting insides using hourly human landing catches (HLC) conducted from 18:00–0:00 h for 3 months. This trial was performed close to the shore of the Lake Abaya to minimize the interference of other animals on mosquito movement. The overnight activity of malaria vectors was assessed within a Chano village from 18:00–6:00 h with collections carried out both indoors and outdoors by HLC. Generalized estimating equations were used to statistically assess differences.

**Results:**

*Anopheles pharoensis* was significantly more prevalent when a calf was present either inside (42%, *P *< 0.001), or adjacent to (46%, *P* = 0.002) a tent relative to a tent without a calf present. The presence of a calf did not affect densities of the primarily anthropophilic species *A. gambiae* (*s.l.*), or *An. tenebrosus*. *Anopheles gambiae* (*s.l.*) (*P *< 0.001) and *An. pharoensis* (*P* = 0.015) both had a tendency for early evening biting between 19:00 h and 20:00 h. *Anopheles gambiae* (*s.l.*) was mainly biting humans outdoors in the village.

**Conclusions:**

The presence of calves within and close to human dwellings acts to draw malaria mosquitoes toward the human occupant with the potential to increase their risk of malaria. Hence, deployment of cattle far from human residence could be recommended to reduce human exposure. Outdoor and early evening biting could threaten the success of current indoor-based interventions. Hence, tools could be designed to reduce this threat.
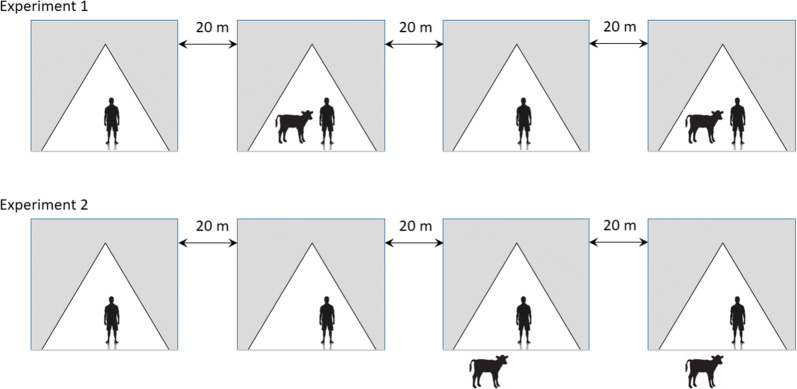

## Background

Malaria is a mosquito-borne disease transmitted through the bites of infected female *Anopheles* mosquitoes [1]. It is prevalent in the tropical and subtropical regions of the world. Since 2000, it is reducing in many malaria-endemic countries due to the combined efforts of distribution of long-lasting insecticidal nets (LLINs), effective case management with effective anti-malarial drugs, larval source management (LSM) and indoor residual spraying (IRS) [2]. Indoor residual spraying and long-lasting insecticidal nets are highly successful where the major vectors are predominantly anthropophilic and endophagic. They are less successful in areas where malaria vectors are exophagic and exophilic [3]. *Anopheles arabiensis*, the principal malaria vector in Ethiopia, shows flexibility with respect to feeding and resting habits, and seems an opportunistic host feeder [4]. It exhibits both anthropophagic and zoophagic behaviors [5]. The other important malaria vector in Ethiopia is *Anopheles pharoensis* which exhibits mostly exophagic behaviour [6].

In areas where malaria vectors have a stronger tendency to bite outdoors or in the early evening when most people are outdoors, residual transmission contributes a greater proportion of all transmission [7]. Residual transmission is any transmission still happening when LLINs and IRS are distributed optimally [8], which highlights the need for new strategies in vector control [9]. It is obvious that the host-seeking and hourly biting activity of malaria vectors are key in malaria vector control programmes.

In southwest Ethiopia people and cattle live in proximity to human dwellings. These livestock may increase human exposure to bites of malaria vectors by attracting mosquitoes towards human dwellings or reduce the human exposure by diverting mosquitoes away from the human hosts. This zoophagic behaviour of vectors may open an opportunity to target animals to tackle residual malaria due to zoophilic vectors. Moreover, if there is a shift in biting hours of the malaria vectors, it may demand supplementary interventions to protect people in the early hours of the day. The present study tests the proximity of calves to people and the effect that this has on the risk of being bitten from an *Anopheles* mosquito that has the potential to transmit malaria. Two experiments were conducted to assess if the presence of a calf either inside or beside a tent where a person spends the night acts to increase or decrease the potential *Anopheles* mosquito bites received. Further analysis was carried out to determine the indoor and outdoor biting activity of malaria vectors in a village adjacent to the experimental site in Arba Minch district, southwest Ethiopia.

## Methods

### Description of the study area

The study was conducted in the Arba Minch district in the Southern Nation, Nationalities and Peoples’ Regional state (SNNPR) of Ethiopia. The study village (Chano Mille village) is about 16 km north of Arba Minch and 470 km south of Addis Ababa. The village is located (at the centre) at 6° 6′ 666″ N longitude and 37° 35′ 775″ E latitude. The altitude of the village is 1206 meters above sea level (masl) at the centre. Malaria is endemic in most villages of the Arba Minch district. The study village is one of the malaria-endemic villages in the district. The climate is hot and humid which is suitable for malaria vectors. The inhabitants are a mix of subsistence farmers. They grow cash crops such as mangoes and bananas as the main source of income. Indoor residual spraying and long-lasting insecticidal nets are the principal malaria vector control tools. *Anopheles arabiensis* is the principal malaria vector in the study area [10].

### Study design

An experimental study was conducted to investigate the host-seeking behavior of malaria vectors. The first experiment was conducted on the shore of Lake Abaya with a high potential for mosquito breeding from October to December 2016. Four tents made of nylon were placed close to the shore and each tent had a single mosquito entry point which can be closed and opened by a zip. The tents were closed during the day to prevent damage by wind, and were left open during the mosquito collection hours (18:00–00:00 h) to allow mosquito entry. Mosquito collection was carried out until midnight due to high wind waves after this time. The lake shore was an open area specifically selected to provide safety against wild animals and minimize diversion of mosquitoes to wild animals for biting.

**Fig. 1:**
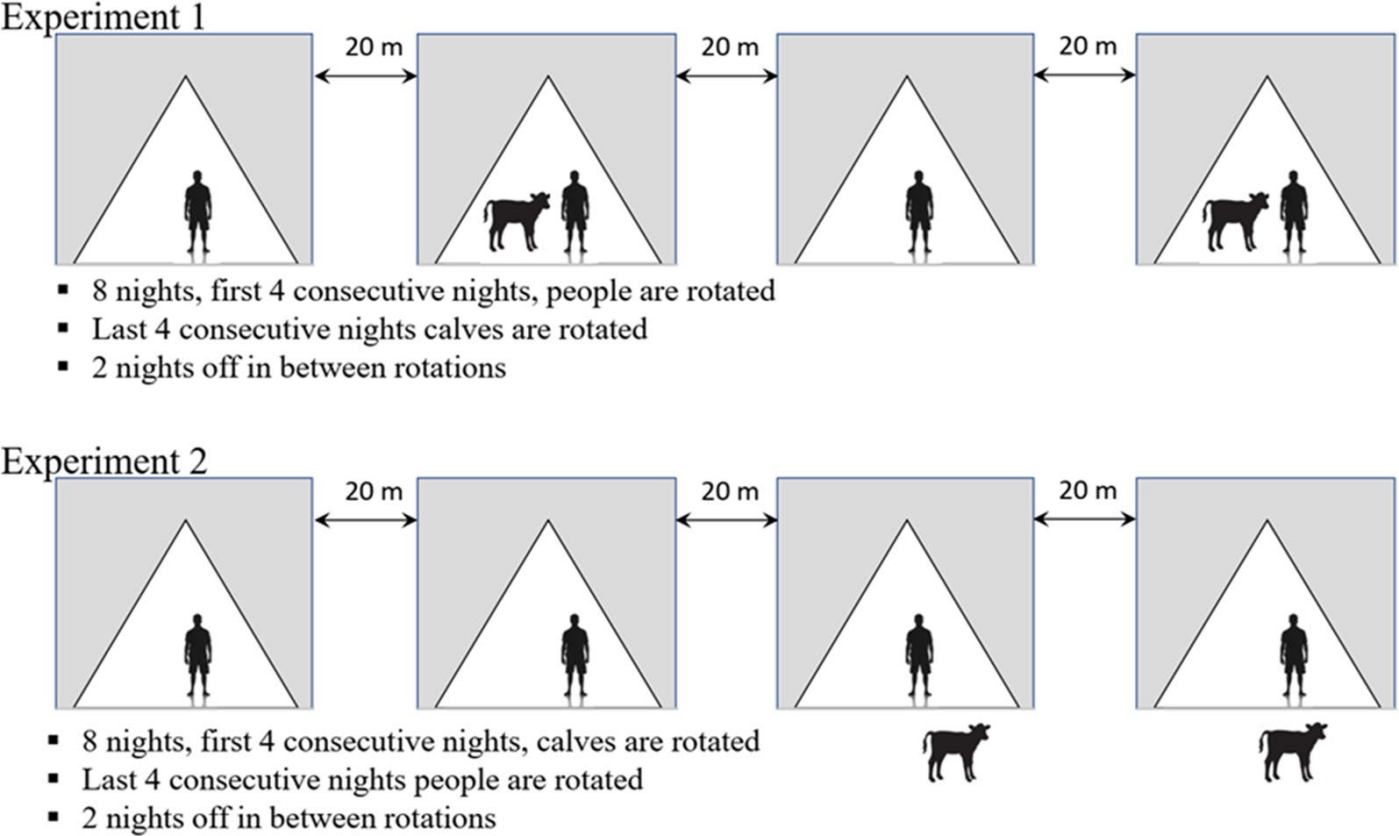
Schematic representation of the study design of the two tent experiments at the shore of the Lake Abaya in Chano Mille, south-west Ethiopia

Four tents were set by the shore of Lake Abaya at 20 m from each other. Four adult male volunteers who had collection experience, and had given written consent were recruited for adult mosquito collection. Two of the tents contained one calf and one adult male volunteer each, and the other two tents contained one adult male volunteer each (Fig. 1). The volunteers in each of the tents performed human landing catches from 18:00 to 0:00 h. Each collector exposed their legs from foot to knee and caught lading mosquitoes using an aspirator. Hourly collected mosquitoes were placed in a separate paper cup.

The rotation of the collectors was done to minimize any bias due to variation in attractiveness and collection skill of individuals, and variation due to the tent location. In the first four consecutive nights, collectors were rotated between tents daily. In the next four consecutive nights, calves were rotated. The whole cycle was completed in eight nights. There were two nights off in between rotations. A total of 32 human landing collections were completed in the first trail.

The second experiment was carried out in the same place. This experimental trial was to test whether the proximity of calves to people increases or decreases the exposure to malaria mosquitoes. One volunteer sat inside each of the four tents. In two of the four tents one calf was placed outside the tent at a distance of 1 m away from the tent (Fig. 1). The volunteers in each of the tents performed human landing catches from 18:00 to 0:00 h. In the first four nights, the calves were rotated. Human collectors were rotated in the following four nights.

The third activity was carried out within the village to assess the biting rhythm and identify the peak biting hours of the malaria vectors. Indoor and outdoor mosquito collection was carried out in two houses with human occupants using HLCs. The two houses were selected randomly. In each house, a pair of collectors interchanged positions indoors and outdoors every hour. The pair of collectors at one house on the first night was rotated to the second house on the next collection night and the cycle continued until the end of the study period, to minimize the bias due to collection skills and attractiveness of the collectors. The distance between the two houses was 200 m. The collection was performed from 18:00 to 6:00 h for seven nights during the study period. Each hour collected mosquitoes were kept separately in a labeled paper cup with date, hour and site of collection. Supervisors frequently visited the collectors to alert them to avoid mosquito bites.

### Identification of *Anopheles* mosquitoes

The collected *Anopheles* mosquitoes were brought to the Medical Entomology Laboratory at Arba Minch University, Arba Minch, Ethiopia. Mosquitoes were killed *via* refrigeration and the species identified by microscopy based on morphological characteristics using an identification key [11]. Then, the identified female *Anopheles* mosquitoes were placed in vials over silica gel for circum-sporozoite protein (CSPs) testing.

### Mosquito processing for CSPs detection

CSP detection was performed by enzyme-link immunosorbent assay (ELISA) [12] at the Arba Minch University Medical Entomology Laboratory. The head and thorax of female *An. pharoensis* was used for *P. falciparum* and *P. vivax*-210 CSP detection. Two separate 96-well micro-titer plates were coated with 50 µl solution of *P. falciparum-* and *P. vivax*-210 monoclonal antibodies (MAB). The plates were covered and incubated overnight at room temperature. Then the contents of the plates were aspirated, emptied and filled with 200 µl blocking buffer (BB) and further incubated for 1 h at room temperature. During the incubation time, mosquitoes were individually grounded in 50 µl grinding solution and the final volume was made up to 250 µl by adding 100 µl BB twice. BB was removed from the plate after 1 h and 50 µl of each homogenized mosquito triturate was added to each of the two test wells. *Plasmodium falciparum-* and *P. vivax*-210-positive samples and a wild-colony of *An. pharoensis* were used as both positive and negative controls. Plates were incubated for 2 h and washed with phosphate-buffered saline (PBS)-Tween 20 twice. Horseradish peroxidase-conjugated monoclonal antibody was then added to each well and incubated for 1 h, and the wells were washed three times with PBS-Tween 20. Finally, 80 µl of peroxidase substrate was added per well and incubated for 30 min. The wells were observed visually for development of a green color and also their optical density was determined at 414 nm in microplate ELISA reader. Samples which had a green color and those with optical values of greater than twice the average optical density of the negative controls were considered positive.

### Outcome variables

The first primary outcome variable was the number of *Anopheles* mosquitoes collected from calf-baited and un-baited human tents, and the number of mosquitoes attempting to bite a human per hour. The secondary outcome variable was the species composition of the *Anopheles* mosquitoes.

### Data analysis

A generalised estimating equation (GEE) model with a negative binomial distribution was used to assess the impact of proximity of animal to human mosquito bite exposure (using SPSS software version 20). A GEE was fitted to the counts of mosquitoes found in either a calf baited or un-baited tent (experiment 1), or to counts of mosquitoes found in a tent with a calf outside or not (experiment 2). The two experiment types were analysed separately. The GEE was used to account a serial correlation between repeated sampling made during each night and the replicate tents. The mean ratio of the number of *Anopheles* species collected from calf baited and un-baited human tents was used to determine the host preference of mosquitoes and the impact of the proximity of the cattle on human exposure. The *Anopheles* mosquito peak biting time was also determined by fitting a GEE with negative binomial distribution to the hourly number of mosquitoes collected. A *P*-value of< 0.05 was used to determine significance in the peak biting hours and the feeding tendency of malaria mosquitoes.

## Results

### Species composition of *Anopheles* mosquitoes

A total of 1593 *Anopheles* mosquitoes belonging to three species were collected during the tent experiment: *An. gambiae* (*s.l.*), *An. pharoensis* and *An. tenebrosus. Anopheles pharoensis* was the dominant species that accounted for 51.4% (819/1593) of the collected mosquitoes, followed by *An. gambiae* (*s.l.*) (34.5%; 550/1593) and *An. tenebrosus* (14.1%; 224/1593). About 174 *An. gambiae* (*s.l.*) were collected indoors and outdoors in Chano village using HLCs.

### Host-seeking tendency of *Anopheles* mosquitoes inside tents with calves

A total of 463 *An. pharoensis* were collected in the first experimental trial. Of these, 284 (61.3%) were collected from tents baited with cattle and 179 (38.7%) were from human tents with no calves. The mean number of *An. pharoensis* was 3.2 in cattle-baited tent/human/night (Wald 95% CI: 2.4–4.2) compared to 1.82 in human tent/night (Wald 95% CI: 1.51–2.2) which was statistically significant (*P* = 0.001).

Of the 421 *An. gambiae* (*s.l.*) collected, 235 (55.8%) were from human tents and 186 (44.2%) were from cattle-baited tents. More *An. gambiae* (*s.l.*) and *An. tenebrosus* were collected from human tents, but it was not statistically significant compared to the cattle-baited tents (Table 1).

**Table 1:** Host-seeking behaviour of *Anopheles* mosquitoes in human tents baited with calf and without calf close to the shore of the Lake Abaya in Chano Mille, south-west Ethiopia

Species	Tent with human, calf baited inside		Tent with human, no calf inside		Mean ratio	% variation	*P*-value
	*n*	Mean (Wald 95% CI)	*n*	Mean (Wald 95% CI)			
*An. pharoensis*	284	3.16 (2.37–4.2)	179	1.82 (1.51–2.2)	0.58 (1.82/3.16)	42	0.001
*An. gambiae* (*s.l*.)	186	2.08 (1.52–2.85)	235	2.43 (1.64–3.59)	0.86 (2.08/2.43)	14	0.500
*An. tenebrosus*	79	0.9 (0.69–1.18)	89	0.82 (0.59–1.14)	0.91 (0.82/0.91)	9	0.340

### Host-seeking behavior of *Anopheles* mosquitoes when calves are outside the tents

Of the 356 *An. pharoensis* collected, 230 (64.6%) were caught from inside human tents where calves were outside the tent at 1 m distance, and 126 (35.4%) were from inside tents where there was no calf outside the tent. Of the 129 *An. gambiae* (*s.l.*) collected, 68 (52.7%) were collected inside the human tents where a calf was kept outside the tent at a distance of 1 m, and 61 (47.3%) were collected inside the human tents where no cattle were kept outside the tents. Though more *An. gambiae* (*s.l.*) (*P* = 0.8) and *An. tenebrosus* (*P* = 0.6) were collected inside human tents with caves outside the tent at 1 m distance, the variation was not statistically significant (Table 2).

**Table 2:** Host-seeking behaviour of *Anopheles* mosquitoes inside tents with a human by keeping the calf at 1-m distance and without calf, at the shore of the Lake Abaya in Chano Mille, south-west Ethiopia

Species	Tent with human, calf outside tent at 1 m		Tent with human, no calf outside tent				
	*n*	Mean (Wald 95% CI)	*n*	Mean (Wald 95% CI)	Mean ratio	% variation	*P*-value
*An. pharoensis*	230	2.42 (1.83–3.10)	126	1.31 (1.02–1.73)	0.54 (1.31/2.42)	46	0.002
*An. gambiae* (*s.l*.)	68	0.71 (0.49–1.02)	61	0.66 (0.38–1.14)	0.93 (0.66/0.71)	7	0.800
*An. tenebrosus*	30	0.3 (0.17–0.53)	26	0.26 (0.16–0.42)	0.87 (0.26/0.30)	13	0.600

### Hourly biting activities of *Anopheles* mosquitoes at the shore of Lake Abaya

**Fig. 2:**
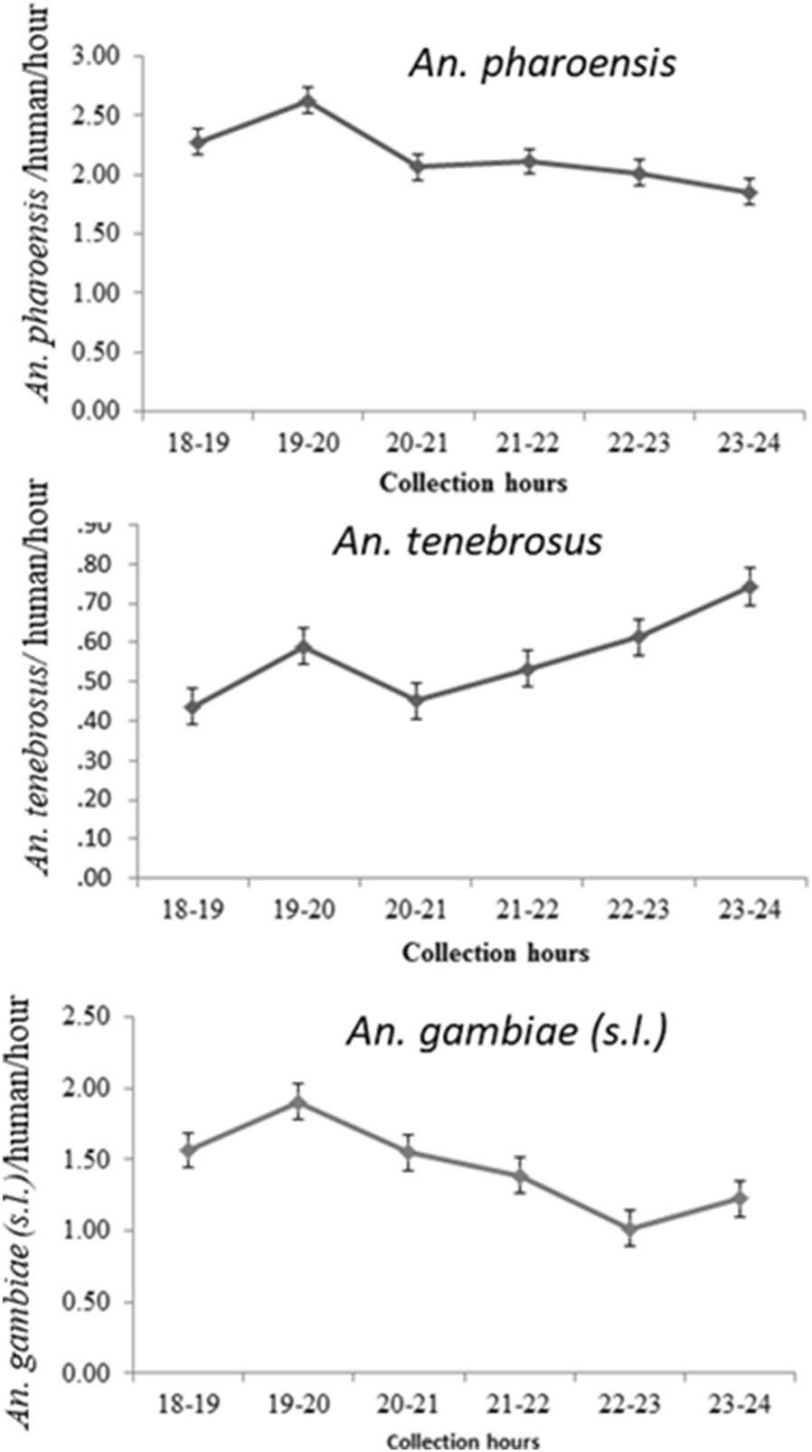
The overall hourly biting activities of the three *Anopheles* species at the shore of Lake Abaya in Chano Mille, south-west Ethiopia

*Anopheles pharoensis* and *An. gambiae* (*s.l.*) showed similar biting activities at the early hours of the night, with peak biting activities between 19:00 and 20:00 h (Fig. 2). Significantly higher numbers of *An. pharoensis* (*P* = 0.015) and *An. gambiae* (*s.l.*) (*P* = 0.0009) were biting humans within the early hours (19:00–20:00 h) close to the breeding sites.

### Indoor and outdoor hourly biting activity of *Anopheles* mosquitoes

**Fig. 3:**
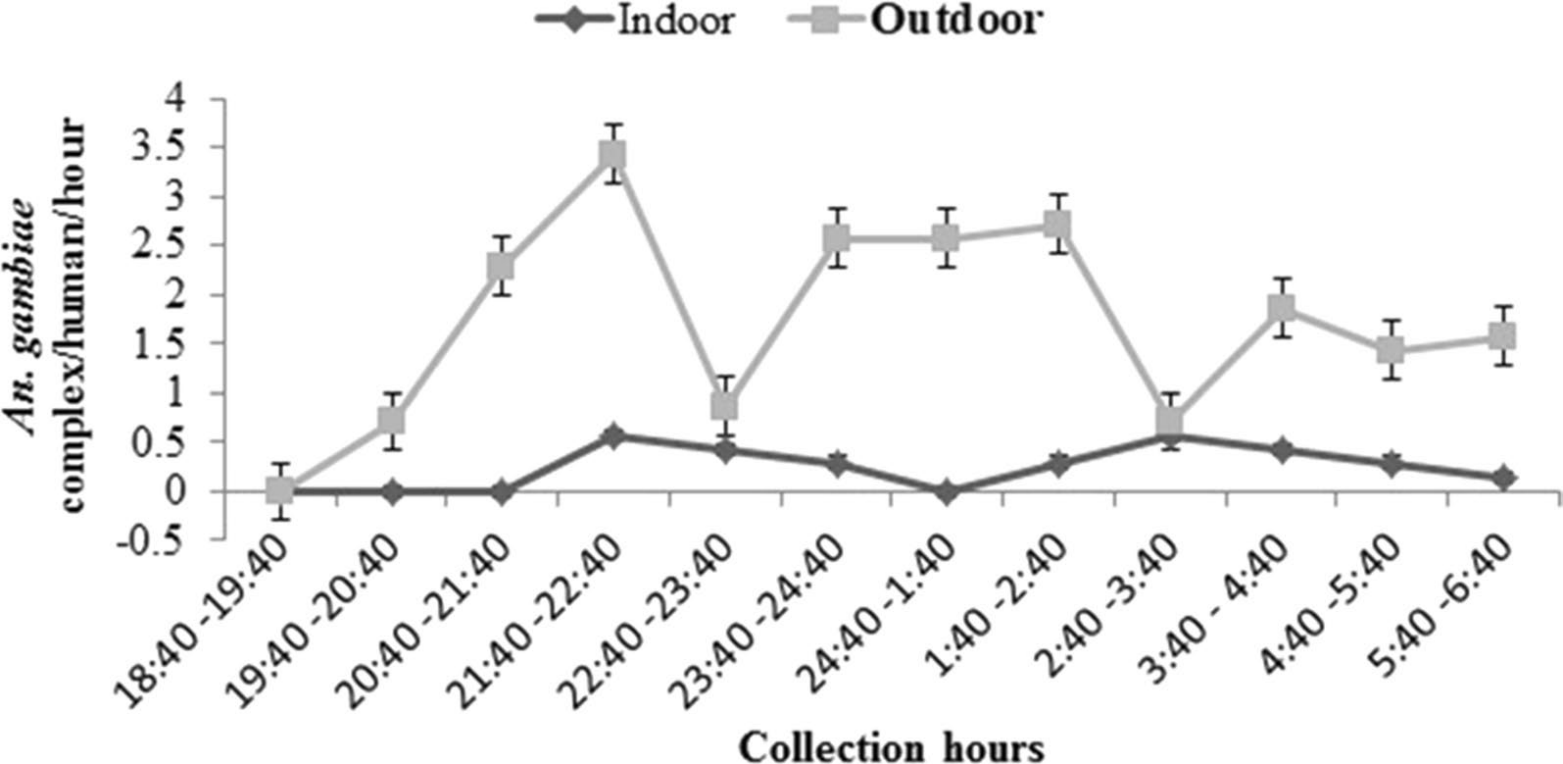
The overnight hourly indoor and outdoor biting activities of *Anopheles gambiae* (*s.l.*) inside the Chano Mille village, south-west Ethiopia

The principal malaria vector *An. gambiae* (*s.l.*) (the sole species collected) showed outdoor biting activities throughout the night. Its outdoor biting activity peaked within the early hours (21:40–22:40 h) of the night (*P *< 0.001) (Fig. 3). Of the 174 *An. gambiae* (*s.l.*) collected inside the village, 87.9% (153/174) were collected outdoors and the remaining 12.1% (21/174) were collected indoors.

### Sporozoite rate of *Anopheles pharoensis*

A total of 792 *An. pharoensis* were tested for CSPs using the ELISA technique, but none of them were positive for *P. falciparum* or *P. vivax* infection.

## Discussion

The main objective of this study was to investigate the impact of cattle on the exposure of humans to host-seeking malaria mosquitoes and the hourly biting rhythm of *Anopheles* mosquitoes. The presence of cattle with a human inside the tent increased the number of *An. pharoensis* by 42% compared to un-baited human tents. The presence of cattle inside a tent where a person spends the night increased the potential *Anopheles* mosquito bites received. Moreover, keeping cattle outside the tent at a distance of at least 1 m from the tent increased the number of *An. pharoensis* inside the tents by 46% compared to human tents with no cattle outside the tent. This implies that the proximity of calves to people and the effect that this has on the risk of being bitten by *Anopheles* mosquito vectors could impact the potential for malaria transmission. This is particularly the case here because, although the zoophilic mosquitoes were not found to have sporozoites, published evidence shows that *An. pharoensis* can be sporozoite-positive [13, 14]. Similarly, the presence of animals and humans in the same house increased biting from malaria vectors (pulling potential of animals) whereas those people living in separate sheds experienced reduced the human biting rates of malaria mosquitoes [15]. On the contrary, the proximity of cattle to human dwellings diverting host-seeking mosquitoes, may provide protection against the bites of *An. arabiensis*. Therefore, for effective uses of zooprophylaxis, livestock should be kept far from human dwellings at night during peak vector activity [16]. Insecticidal zooprophylaxis would also have an impact on malaria vector density by inducing mortality when feeding on insecticide-treated animals [17]. Zooprophylaxis, the use of animals to divert malaria vectors away from humans could be used to control those mosquitoes attracted to animals [18], though the results of zooprophylaxis are controversial; some claim that animals reduce malaria infection and the other claim the opposite [18, 19]. Hence, we propose the use of insecticides for treatment of animals to kill those vectors attracted by the animals.

In the present study, the majority of *Anopheles* species were biting humans mainly during the early hours of the night, when most people are not yet beneath bednets. The outdoor and early hours biting tendency of malaria vectors has been documented in south-central Ethiopia [6]. A study in south-west Ethiopia also reported an early hour’s biting tendency of *An. pharoensis* [20]. We also documented the outdoor biting tendency of *An. gambiae* (*s.l.*) throughout the night with a peak biting activity during early hours in the village, where IRS and LLINs are the cornerstone interventions implemented. On the other hand, a study from Sille in Ethiopia in 2006 documented a different biting pattern of *An. arabiensis*, with peak biting activity occurring during the late hours of the night [20]. The outdoor biting behaviour of *An. gambiae* (*s.l.*) could compromise the efficacy of the indoor-based key interventions IRS and LLINs. These behaviours could also result in persistence of residual malaria transmission, even after high coverage and use of IRS and LLINs [8]. Hence, vector control interventions such as insecticidal zooprophylaxis could be implemented to target outdoor and early hours biting vectors [21] in an area where animals usually stay outdoors at night.

During this study period, three *Anopheles* species, namely *An. gambiae* (*s.l.*), *An. pharoensis* and *An. tenebrosus* were recorded. *Anopheles pharoensis* was the dominant species on the shores of the Lake Abaya; this is due to the presence of permanent water and grasses that grow near to the lakeshore during the collection period. This is the ideal place for *An. pharoensis* breeding, and small water bodies created by hoof prints of cattle and hippopotami make the lakeshore a potential mosquito breeding site. This species prefers breeding habitats with permanent water bodies and vegetation and is widely distributed in Ethiopia [6, 14, 20]. No sporozoite-infected *An. pharoensis* were recorded. This could be due to the collection of mosquitoes close to breeding sites where younger mosquitoes might be dominant.

This study had several strengths and limitations. The HLC method was used for collecting mosquitoes which is the gold standard to estimate the host biting behavior of malaria vectors. Rotation of human collectors and hosts is believed to minimize the bias due to attraction and spatial variation. The tent experiment was conducted at the shore of the Lake Abaya where the density of mosquitoes was high and there was no interference of other animals. The identification of mosquitoes was achieved using morphological identification keys, which may have led to misclassification. The human volunteers may have missed some mosquitoes during collection and when transferring them into paper cups, and this may bias the estimation of the mosquito biting hours.

## Conclusions

The presence of cattle and humans inside the tent increased the number of malaria vectors biting humans. Keeping cattle close to the human tent increased the number of malaria vector bites on humans. Hence, livestock should be kept far from human dwellings at night during peak vector activity. The principal malaria vector, *An. gambiae* (*s.l.*), bites humans mainly outdoors and in the early hours of the night when people are not protected by bednets. Finally, outdoor and early hours biting behavior of the vectors could threaten the effectiveness of current indoor-based interventions and hence, new tools should be designed. Moreover, deployment of cattle far from human residence may be recommended to reduce human exposure to malaria vectors.

## Data Availability

The data supporting the conclusions of this article is presented within the article.

## Abbreviations

LLINs: long-lasting insecticidal nets
IRS: indoor residual spraying
ELISA: enzyme-linked immunosorbent assay
CSPs: circum-sporozoite protein
LSM: larval source management
SNNPR: southern nation, nationalities and peoples’ regional state
HLCs: human landing catches
